# Clinical efficacy of dupilumab in the treatment of severe chronic rhinosinusitis: The first case outside of a clinical trial

**DOI:** 10.1002/ccr3.3792

**Published:** 2021-01-16

**Authors:** Matteo Trimarchi, Pietro Indelicato, Alessandro Vinciguerra, Mario Bussi

**Affiliations:** ^1^ Otorhinolaryngology Unit Division of Head and Neck Department IRCCS San Raffaele Scientific Institute Milano Italy; ^2^ School of Medicine Vita‐Salute San Raffaele University Milano Italy

**Keywords:** anti–IL‐13, anti–IL‐4, biologic agents, chronic rhinosinusitis, dupilumab, DUPIXENT, monoclonal antibody, nasal polyposis, type 2 inflammation

## Abstract

Treatment options for severe CRSwNP are limited. Dupilumab is a safe, well‐tolerated, and effective alternative in patients with poor control of symptoms, corticosteroid‐dependent disease, and high rates of recurrence of nasal polyps after surgery.

## Graphical abstract

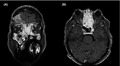

## 1 INTRODUCTION

Chronic rhinosinusitis with nasal polyps is a type 2–mediated inflammatory disease associated with significant burden due to symptoms and high recurrence rate after surgery. Dupilumab, a monoclonal antibody against the interleukin‐4 receptor subunit α, has demonstrated good clinical efficacy and acceptable safety in phase II and phase III trials.

Chronic rhinosinusitis with nasal polyps (CRSwNP) is a complex inflammatory disorder of the upper airways affecting approximately 4.3% of the population in Europe^1^ and defined clinically by symptoms of nasal congestion, discharge, facial pressure, loss of smell, and postnasal drip lasting more than 12 weeks.^2^ CRSwNP is characterized by T‐helper type 2 cell (Th2) inflammation, with marked infiltration of eosinophils and mast cells in nasal mucosa and polyp tissue, which leads to both local and systemic increases in the levels of type 2 cytokines, including eosinophil cationic protein (ECP), eotaxin, interleukin (IL)‐4, IL‐5, and IL‐13.^3^, ^4^, ^5^ Type 2 cytokines such as IL‐4, IL‐5, and IL‐13 also play an important role in pathophysiological mechanisms of CRSwNP‐associated diseases such as aspirin‐exacerbated respiratory disease (AERD) and asthma.^6^, ^7^ In particular, up to two‐thirds of patients with CRSwNP are affected by comorbid asthma, resulting in more severe nasal obstruction, higher rates of recurrence of nasal polyps after surgery, poor asthma control, and significant impairment of quality of life (QoL).^8^, ^9^, ^10^, ^11^

Dupilumab (DUPIXENT®) is a fully human, VelocImmune‐derived^12^, ^13^ IgG‐4 monoclonal antibody that blocks the shared receptor subunit of IL‐4 and IL‐13, thus inhibiting signaling of both IL‐4 and IL‐13.^14^ Subcutaneous administration of dupilumab, alone or in combination with topical corticosteroids, has demonstrated good clinical efficacy in patients with moderate‐to‐severe asthma with an eosinophilic phenotype^15^ and in the treatment of atopic dermatitis.^16^ Importantly, dupilumab is the first monoclonal antibody approved by the FDA and EMA as add‐on therapy to intranasal corticosteroids in adult patients with severe CRSwNP and not adequately controlled by medical and surgical treatments.^17^

Herein, we report the first case of a patient with recalcitrant and severe CRSwNP who has been treated with dupilumab outside the framework of a clinical trial.

## 2 CASE PRESENTATION

A 65‐year‐old man visited our department complaining of recurrent nasal obstruction, facial pain, and loss of smell despite ongoing treatment with oral corticosteroids. Over the last 10 years, the patient had taken oral corticosteroids and antibiotics every 2‐3 months for multiple acute exacerbation of CRS. Furthermore, he had undergone 7 functional endoscopic sinus surgeries (FESS) and 2 osteoplastic frontal sinusotomies for recalcitrant CRSwNP, and the last was complicated by a bilateral frontal abscess with intracranial epidural extension, which required additional neurosurgical intervention. In addition, the patient was diagnosed with asthma and was chronically treated with a combination of a long‐acting beta‐agonist (LABA) and inhaled corticosteroids (ICSs).

**FIGURE 1:**
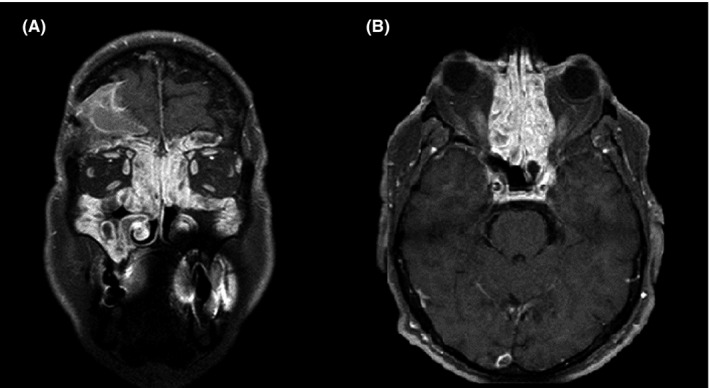
Coronal (A) and axial (B) CT scan performed before dupilumab treatment. Imaging shows a massive recurrence of nasal polyposis resulting in complete obstruction of the maxillary, frontal, and ethmoidal paranasal sinuses

At admission to our department, nasal endoscopy revealed a massive recurrence of nasal polyposis, with a bilateral endoscopic nasal polyp score (NPS) of 5, resulting in olfactory cleft obliteration and subtotal nasal obstruction. University of Pennsylvania Smell Identification Test (UPSIT) and Sino‐Nasal Outcome Test (SNOT‐22) revealed scores of 9 and 43, respectively. Maxillofacial computed tomography (MXF‐CT) and magnetic resonance imaging (MRI) confirmed the relapse of nasal polyposis occupying the nasal cavity and obstructing the maxillary, frontal, and ethmoidal paranasal sinuses. (Figure 1) Considering the history of multiple nasal surgeries, the repeated courses of oral corticosteroids, the loss of smell, and a diagnosis of comorbid asthma, the patient was indicated to receive dupilumab, in accordance with the European Forum for Research and Education in Allergy and Airway Disease (EUFOREA) consensus on biologics for CRSwNP.^18^

To our knowledge, this is the first case report outside the framework of a clinical trial of a patient affected by CRSwNP and comorbid asthma, for whom dupilumab was mainly chosen for the severity of his nasal pathology.

**FIGURE 2:**
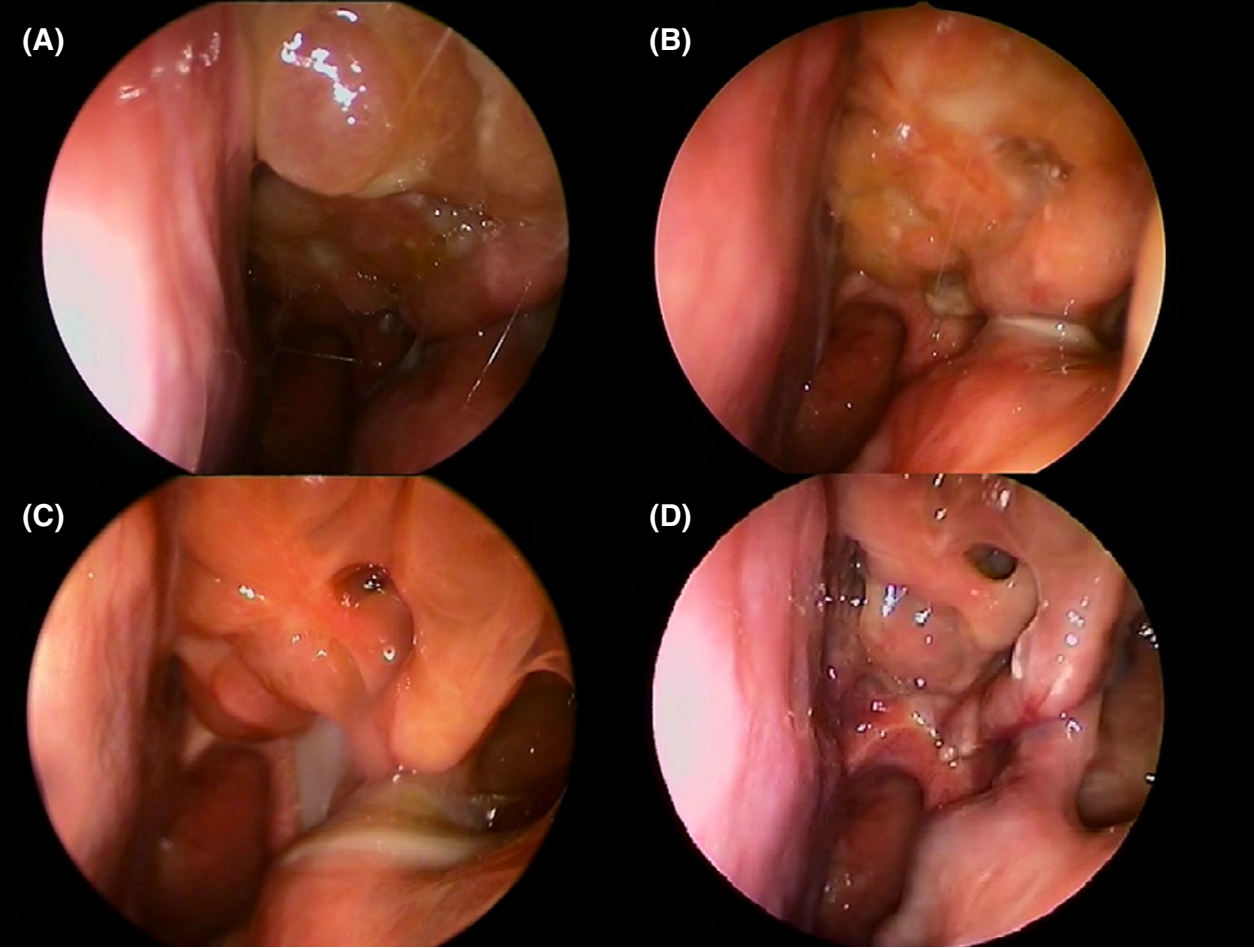
Nasal endoscopy prior to starting subcutaneous dupilumab (A) and after 1 (B), 3 (C), and 6 mo (D) of treatment

**FIGURE 3:**
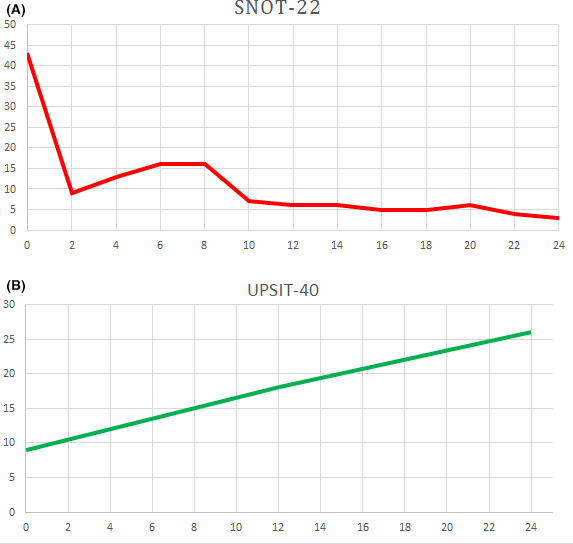
Trends of SNOT‐22 (A) and UPSIT scores (B)

Starting from April 2020, one dose of 300 mg of subcutaneous dupilumab was administered every 2 weeks for 6 months (26 weeks). SNOT‐22 and nasal endoscopy were assessed during each follow‐up visit, and the UPSIT score was assessed at the beginning, middle, and end of treatment. Three months after the initiation of dupilumab, the patient reported significant relief in nasal symptoms with a change in UPSIT and SNOT‐22 scores (18 vs 9 and 6 vs 43, respectively); nasal endoscopy demonstrated a reduction in the size of bilateral polyposis (NPS 4 vs 5). No significant side effects were observed. Six months after treatment, a clinically meaningful control of nasal symptoms was reached, confirmed by improvement in the findings of nasal endoscopy (Figure 2) and by partial but persistent recovery of the loss of smell (UPSIT score 25, SNOT‐22 score 3; Figure 3). There were no exacerbations of asthma during treatment with dupilumab.

## 3 DISCUSSION

CRSwNP is a severe subtype of chronic rhinosinusitis generally characterized by a high burden due to symptoms, as well as frequent recurrence of nasal polyps, loss of smell, asthma comorbidity, and poor health‐related QoL.^11^ Treatments for severe CRSwNP are limited. Inhaled corticosteroids are associated with minimal benefits, and repeated courses of oral corticosteroids may lead to long‐term side effects such as steroid‐induced diabetes and osteoporosis; sinus surgery is frequently burdened by high rates of recurrence.^19^, ^20^, ^21^ Recent therapeutic approaches have focused on controlling the underlying mucosal inflammatory process, referred to as “type 2 inflammation,” which is characterized by massive tissue eosinophilia, T‐helper type 2 cell infiltration, and increased levels of the type 2 cytokines IL‐4, IL‐5, and IL‐13.^22^, ^23^, ^24^, ^25^

By specifically binding and blocking the α subunit of the IL‐4 receptor (IL‐4Rα), which is shared by IL‐4 and IL‐13, the fully human monoclonal antibody dupilumab has been demonstrated to be effective and generally well‐tolerated in other type 2–related diseases.^16^, ^26^, ^27^, ^28^, ^29^

The efficacy of dupilumab in CRSwNP was first evaluated in a randomized double‐blinded, placebo‐controlled, phase II study carried out in 2016 by Bachert and colleagues.^30^ After a 4‐week run‐in of treatment with mometasone furoate nasal spray, 60 adult patients with CRSwNP were randomly allocated to add‐on therapy with subcutaneous dupilumab (600 mg loading dose followed by 15 weekly doses of 300 mg) or placebo for 16 weeks. Patients treated with dupilumab had significant improvement in SNOT‐22, UPSIT smell score, endoscopically graded nasal polyp score (NPS), and Lund‐Mackay sinus (LMS) CT score. In addition, dupilumab improved lung function (as assessed by FEV_1_, forced expiratory volume in 1 second) and asthma control (as assessed by ACQ‐6, asthma control questionnaire) in the subgroup of patients with comorbid asthma.^30^

Considering the positive results obtained in the phase II study, phase III trials were carried out. In two randomized double‐blind, multicenter, placebo‐controlled, parallel‐group studies (SINUS‐24 and SINUS‐52), Bachert et al^17^ evaluated the efficacy and safety of dupilumab added to standard of care in adults with severe CRSwNP. In SINUS‐24, patients were randomized 1:1 to 24 weeks of subcutaneous dupilumab 300 mg or placebo every two weeks, while in SINUS‐52, patients were randomized 1:1:1 to 52 weeks of dupilumab 300 mg every two weeks, 24 weeks every two weeks and then 28 weeks of dupilumab 300 mg every four weeks, or 52 weeks of placebo every two weeks. In both studies, treatment with dupilumab significantly improved SNOT‐22, UPSIT smell score, NPS, LMS, and asthma outcomes (FEV_1_ and control) compared with placebo.^17^

Importantly, the aforementioned studies highlighted the efficacy of dupilumab in both the overall population and the subset of patients with higher disease burden, composed of patients who had previously undergone multiple nasal surgeries or with comorbid asthma, which has been reported to be a risk factor for postoperative recurrence of nasal polyps.^31^, ^32^ In a study by Mendelsohn and colleagues, recurrence rates of nasal polyposis at 5 years were 16%, 45%, and 90% for control patients, patients with asthma, and patients with Samter's triad, respectively, with rates of revision surgery at 5 years of 10%, 25%, and 37%, respectively.^33^

Our patient suffered from a severe form of CRwNP, characterized by a high rate of recurrence, unresponsiveness to topical and systemic medical treatment, and comorbidity with asthma, resulting in significant impairment of QoL. Considering the inefficacy of previous endoscopic or external surgical treatment and since the patient fulfilled the indications of the EUFOREA consensus on biologic agents for CRSwNP,^18^ we decided to not perform additional surgery, but to treat the patient with dupilumab.

Even if the results of dupilumab in phase II and phase III trials are promising, there are some unresolved questions that need to be answered. First, studies on dupilumab in CRSwNP have been limited from 16 to 52 weeks of treatment; no data on long‐term efficacy or on the schedule, duration, or dosage for maintenance therapy are currently available. Second, not all the patients included in the clinical trials above mentioned fulfilled the criteria of type 2 inflammation (tissue eosinophils ≥ 10/high‐power field or blood eosinophils ≥ 250/µL or total IgE ≥ 100 IU/mL).^2^ A future pathophysiological‐based selection of patient candidate to biologic agents may be related to better outcomes in “real‐life” clinical practice. Third, biologics are expensive and involve multiple physician visits. Considering the high costs of treatment, future studies need to be focused on the identification of biologic markers that may predict individual treatment response. Finally, to date there is no consensus on the position of biologic agents in the therapeutic algorithm of CRSwNP. Specifically, it is not clear whether dupilumab may be administered preoperatively, postoperatively, alternatively to surgery, or in case of surgical failure.

In conclusion, dupilumab is safe, well‐tolerated, and clinically effective in adults with severe CRSwNP and in the group of patients with high burden of symptoms and in whom conventional medical and surgical treatment had failed, such as the case we present herein. Future studies need to address the optimal utilization of this promising therapeutic weapon.

## CONFLICT OF INTEREST

The authors declared no potential conflicts of interest with respect to the research, authorship, and/or publication of this article.

## AUTHOR CONTRIBUTIONS

Matteo Trimarchi: conceptualized and designed the study, revised the manuscript critically for important intellectual content, and approved the final version. Pietro Indelicato: drafted the article and made substantial contributions to the acquisition of data. Alessandro Vinciguerra: drafted the article and made substantial contributions to the acquisition of data. Mario Bussi: conceptualized and designed the study, revised the manuscript critically for important intellectual content, and approved the final version.

## ETHICAL STATEMENT

We obtained a formal approval from AIFA (Agenzia Italiana del Farmaco) considering the recent authorization by the FDA and EMA of dupilumab treatment in adult patients with severe CRSwNP. We received informed consent from the patient for all the procedures described in this case report. The study was conducted according to the ethical standards established in the Declaration of Helsinki.

## Data Availability

Data pertaining to this article are available from the corresponding author upon reasonable request.

## References

[1] Hedman J , Kaprio J , Poussa T , Nieminen MM . Prevalence of asthma, aspirin intolerance, nasal polyposis and chronic obstructive pulmonary disease in a population‐based study. Int J Epidemiol. 1999;28(4):717‐722. 10480701 10.1093/ije/28.4.717

[2] Fokkens WJ , Lund VJ , Hopkins C , et al. European Position Paper on Rhinosinusitis and Nasal Polyps 2020. Rhinology. 2020:1‐464. 10.4193/Rhin20.600 32077450

[3] Gröger M , Bernt A , Wolf M , et al. Eosinophils and mast cells: A comparison of nasal mucosa histology and cytology to markers in nasal discharge in patients with chronic sino‐nasal diseases. Eur Arch Oto‐Rhino‐Laryngology. 2013;270(10):2667‐2676. 10.1007/s00405-013-2395-2 23430080

[4] Van Zele T , Claeys S , Gevaert P , et al. Differentiation of chronic sinus diseases by measurement of inflammatory mediators. Allergy Eur J Allergy Clin Immunol. 2006;61(11):1280‐1289. 10.1111/j.1398-9995.2006.01225.x 17002703

[5] Van Crombruggen K , Zhang N , Gevaert P , Tomassen P , Bachert C . Pathogenesis of chronic rhinosinusitis: Inflammation. J Allergy Clin Immunol. 2011;128(4):728‐732. 21868076 10.1016/j.jaci.2011.07.049

[6] Philpott CM , Erskine S , Hopkins C , et al. Prevalence of asthma, aspirin sensitivity and allergy in chronic rhinosinusitis: Data from the UK National Chronic Rhinosinusitis Epidemiology Study. Respir Res. 2018;19(1).129 10.1186/s12931-018-0823-y. 29945606 PMC6020303

[7] Massoth L , Anderson C , McKinney KA . Asthma and chronic rhinosinusitis: diagnosis and medical management. Med Sci. 2019;7(4):53. 10.3390/medsci7040053 PMC6524348 30934800

[8] Bachert C , Zhang L , Gevaert P . Current and future treatment options for adult chronic rhinosinusitis: Focus on nasal polyposis. J Allergy Clin Immunol. 2015;136(6):1431‐1440. 26654192 10.1016/j.jaci.2015.10.010

[9] Orlandi RR , Kingdom TT , Hwang PH . International consensus statement on allergy and rhinology: rhinosinusitis executive summary. Int Forum Allergy Rhinol. 2016;6(S1):S3‐S21. 26878819 10.1002/alr.21694

[10] Bilodeau L , Boulay MÈ , Prince P , Boisvert P , Boulet LP . Comparative clinical and airway inflammatory features of asthma with or without nasal polyposis. Rhinology. 2010;48(4):420‐425. 21442078 10.4193/Rhino09.095

[11] Alobid I , Bernal‐Sprekelsen M , Mullol J . Chronic rhinosinusitis and nasal polyps: The role of generic and specific questionnaires on assessing its impact on patient’s quality of life. Allergy Eur J Allergy Clin Immunol. 2008;63(10):1267‐1279. 10.1111/j.1398-9995.2008.01828.x 18782106

[12] Macdonald LE , Karow M , Stevens S , et al. Precise and in situ genetic humanization of 6 Mb of mouse immunoglobulin genes. Proc Natl Acad Sci U S A. 2014;111(14):5147‐5152. 24706858 10.1073/pnas.1323896111 PMC3986150

[13] Murphy AJ , Macdonald LE , Stevens S , et al. Mice with megabase humanization of their immunoglobulin genes generate antibodies as efficiently as normal mice. Proc Natl Acad Sci U S A. 2014;111(14):5153‐5158. 24706856 10.1073/pnas.1324022111 PMC3986188

[14] Gandhi NA , Pirozzi G , Graham NMH . Commonality of the IL‐4/IL‐13 pathway in atopic diseases. Expert Rev Clin Immunol. 2017;13(5):425‐437. 28277826 10.1080/1744666X.2017.1298443

[15] Deeks ED . Dupilumab: a review in moderate to severe asthma. Drugs. 2019;79(17):1885‐1895. 31728838 10.1007/s40265-019-01221-x

[16] Frampton JE , Blair HA . Dupilumab: a review in moderate‐to‐severe atopic dermatitis. Am J Clin Dermatol. 2018;19(4):617‐624. 30027349 10.1007/s40257-018-0370-9

[17] Bachert C , Han JK , Desrosiers M , et al. Efficacy and safety of dupilumab in patients with severe chronic rhinosinusitis with nasal polyps (LIBERTY NP SINUS‐24 and LIBERTY NP SINUS‐52): results from two multicentre, randomised, double‐blind, placebo‐controlled, parallel‐group phase 3 trials. Lancet. 2019;394(10209):1638‐1650. 31543428 10.1016/S0140-6736(19)31881-1

[18] Fokkens WJ , Lund V , Bachert C , et al. EUFOREA consensus on biologics for CRSwNP with or without asthma. Allergy Eur J Allergy Clin Immunol. 2019;74(12):2312‐2319. 10.1111/all.13875 PMC6972984 31090937

[19] Hopkins C . Chronic rhinosinusitis with nasal polyps. N Engl J Med. 2019;381(1):55‐63. 31269366 10.1056/NEJMcp1800215

[20] Wynn R , Har‐El G . Recurrence rates after endoscopic sinus surgery for massive sinus polyposis. Laryngoscope. 2004;114(5):811‐813. 15126735 10.1097/00005537-200405000-00004

[21] Vlaminck S , Vauterin T , Hellings PW , et al. The importance of local eosinophilia in the surgical outcome of chronic rhinosinusitis: A 3‐year prospective observational study. Am J Rhinol Allergy. 2014;28(3):260‐264. 24980239 10.2500/ajra.2014.28.4024

[22] De Greve G , Hellings PW , Fokkens WJ , Pugin B , Steelant B , Seys SF . Endotype‐driven treatment in chronic upper airway diseases. Clin Transl Allergy. 2017;7(1):22. 28706720 10.1186/s13601-017-0157-8 PMC5506670

[23] Laidlaw TM , Buchheit KM . Biologics in chronic rhinosinusitis with nasal polyposis. Ann Allergy Asthma Immunol. 2020;124(4):326‐332. 31830587 10.1016/j.anai.2019.12.001 PMC7113089

[24] Kartush AG , Schumacher JK , Shah R , Patadia MO . Biologic agents for the treatment of chronic rhinosinusitis with nasal polyps. Am J Rhinol Allergy. 2019;33(2):203‐211. 30587005 10.1177/1945892418814768

[25] Lanzillotta M , Campochiaro C , Trimarchi M , et al. Deconstructing IgG4‐related disease involvement of midline structures: Comparison to common mimickers. Mod Rheumatol. 2017;27(4):638‐645. 27622319 10.1080/14397595.2016.1227026

[26] Rabe KF , Nair P , Brusselle G , et al. Efficacy and safety of dupilumab in glucocorticoid‐dependent severe asthma. N Engl J Med. 2018;378(26):2475‐2485. 29782224 10.1056/NEJMoa1804093

[27] Wenzel S , Ford L , Pearlman D , et al. Dupilumab in persistent asthma with elevated eosinophil levels. N Engl J Med. 2013;368(26):2455‐2466. 23688323 10.1056/NEJMoa1304048

[28] Trimarchi M , Bellini C , Fabiano B , Bussi M , Gerevini S . Multiple mucosal involvement in cicatricial pemphigoid. Acta Otorhinolaryngol Ital. 2009;29(4):222‐225. 20161882 PMC2816372

[29] Trimarchi M , Bondi S , Della Torre E , Terreni MR , Bussi M . Palate perforation differentiates cocaine‐induced midline destructive lesions from granulomatosis with polyangiitis. Acta Otorhinolaryngol Ital. 2017;37(4):281‐285. 28663599 10.14639/0392-100X-1586 PMC5584099

[30] Bachert C , Mannent L , Naclerio RM , et al. Effect of subcutaneous dupilumab on nasal polyp burden in patients with chronic sinusitis and nasal polyposis: A randomized clinical trial. JAMA ‐ J Am Med Assoc. 2016;315(5):469‐479. 10.1001/jama.2015.19330 26836729

[31] Wu C , Lee T , Huang C , Chang P , Fu C . Am J Otolaryngol Clinical predictors of revision surgery for chronic rhinosinusitis with nasal polyposis within 5‐year follow‐up. Am J Otolaryngol. 2020;41(6):102654. 32805665 10.1016/j.amjoto.2020.102654

[32] Trimarchi M , Bellini C , Toma S , Bussi M . Back‐and‐forth endoscopic septoplasty: Analysis of the technique and outcomes. Int Forum Allergy Rhinol. 2012;2(1):40‐44. 22311840 10.1002/alr.20100

[33] Mendelsohn D , Jeremic G , Wright ED , Rotenberg BW . Revision rates after endoscopic sinus surgery: a recurrence analysis. Ann Otol Rhinol Laryngol. 2011;120(3):162‐166. 21510141 10.1177/000348941112000304

